# Editorial: Sex determination and developmental mechanism of crustaceans and shellfish, volume II

**DOI:** 10.3389/fendo.2023.1155209

**Published:** 2023-02-20

**Authors:** Ardavan Farhadi

**Affiliations:** Key Laboratory of Tropical Hydrobiology and Biotechnology of Hainan Province, Hainan Aquaculture Breeding Engineering Research Center, College of Marine Sciences, Hainan University, Haikou, Hainan, China

**Keywords:** crustacean, shellfish, sex-determination, reproduction, steroid hormone

Crustaceans are considered as valuable food sources, due to their high market and nutrition value. The molecular mechanism of sexual development in crustaceans is still unclear and further studies are needed to get a clear understanding and improve their reproductive efficiency. The androgenic gland (AG) plays an important role in sexual development by secretion of insulin-like androgenic gland hormone (IAG) (Farhadi et al., 2021a). The IAG is considered as one of the most important hormones in the sexual development of male crustaceans. Recent studies revealed that sexual development in crustaceans is not that simple and several other hormones and sexual genes are involved in this process. For example, recent studies have found several sexual genes such as *Wnt4*, *CFSH*, *Dmrt gene family*, *Sox gene family*, *Fem-1*, etc. play key roles in the regulation of IAG and sexual development in direct or indirect ways (Farhadi et al., 2021b). Up to now, the complete and functional sex reversal only has been reported in giant freshwater prawn, *Macrobrachium rosenbergii*. Therefore, more studies are needed to discover more sexual factors (i.e., sex-related genes and molecular pathways) in different crustaceans species. Identification and functional analysis of the sex-related genes will help us to find novel techniques for sexual manipulation.

Based on the past successful Research Topic “*Sex Determination and Developmental Mechanism of Crustaceans and Shellfish*”, we are pleased to announce the Volume II. This Research Topic “*Sex Determination and Developmental Mechanism of Crustaceans and Shellfish - Volume II*” gathers four research articles dedicated to the molecular mechanism of sexual development in crustaceans. The articles identified and revealed the function of several key sex-related genes (i.e., *polo-like kinase 1*, *insulin-like peptide*, *steroidogenesis genes*, *LGR1*, *GPA2*, *GPB5*) in different crustacean species.

In oriental river prawn (*Machrobrachium nipponense*), Jin et al. identified and revealed the function of a *polo-like kinase 1 gene* (*Mn-Plk1*) in the sexual development of male *M. nipponense*. The full-length cDNA of *Mn-Plk1* was 2360 bp with an open reading frame (ORF) encoding 611 amino acids. *Mn-Plk1* had the highest expression level in the male and female gonads compared to other tissues, the highest expression level was detected in the male testis. The RNA interference experiment revealed that the knockdown of *Mn-Plk1* decreased the expression of *IAG* in males. Moreover, fewer sperm cells were observed after the dsPlk1 injection. These findings showed that the testis development in *M. nipponense* is regulated by *Mn-Plk1*.

In another study, Wahl et al. identified and characterized three sex-related genes including *GPA2* (*MrGPA2*), *GPB5* (*MrGPB5*), and *LGR1* (*MrLGR1*) in giant freshwater prawn (*Macrobrachium rosenbergii*). The RNAi experiment on female *M. rosenbergii* revealed a negative correlation between *MrGPA2*/*MrGPB5* silencing and *MrLGR1* transcript levels, suggesting a possible ligand-receptor interaction. After *MrGPA2*/*MrGPB5* knockdown, the expression level of the vitellogenin gene was significantly reduced. Moreover, the knockdown of *MrLGR1* increased the expression level of *MrVg receptor* (*MrVgR*) in the ovary, which lead to increasing the size of oocyte cells. These results showing that the *GPA2*/*GPB5* heterodimer acts as a gonad inhibiting factor in the eyestalk-hepatopancreas-ovary endocrine axis in *M. rosenbergii*.

In the blue crab (*Callinectes sapidus*), Wang et al. revealed the role of 17b-estradiol (E2) in the sexual development of female crabs. In this study, several genes related to steroidogenic pathways were identified and characterized including *StAR3*, *3bHSD*, *17bHSD8*, and *ERR*. The expression analysis showed that these genes were widely expressed in several tissues such as hepatopancreas, ovary, eyestalk ganglia, brain, ovigerous, spermathecae, and plumose setae. *In situ* hybridization (ISH) showed that *17bHSD8* transcripts were localized in the follicle cells of the ovary. Furthermore, the injection of CFSH-dsRNA decreased the transcript levels of *E2* and *StAR3*. These findings showed that the mode of *CFSH* action in *C. sapidus* might involve *E2* in these adult-female-specific tissues.

In the ridgetail white prawn (*Exopalaemon carinicauda*), Gao et al. revealed the role of an *insulin-like peptide encoding* (*EcILP*) gene by using gene cloning, expression analysis, RNA interference, and CRISPR/Cas9 genome editing techniques. The multiple sequence alignment, phylogenic tree, and expression analysis revealed that *EcILP* was similar to vertebrate *insulin/IGFs* and insect *ILPs* in its heterodimeric structure and expression profile. The *EcILP* knockout resulted in significantly higher growth-inhibitory traits and mortality. Moreover, the knockdown of *EcILP* caused slow growth and a lower survival rate. These findings showed that *EcILP* could be a key growth regulator in *E. carinicauda*.

## Author contributions

All authors listed have made a substantial, direct, and intellectual contribution to the work and approved it for publication.

